# KCa3.1 mediates dysfunction of tubular autophagy in diabetic kidneys via PI3k/Akt/mTOR signaling pathways

**DOI:** 10.1038/srep23884

**Published:** 2016-03-31

**Authors:** Chunling Huang, Mike Z. Lin, Delfine Cheng, Filip Braet, Carol A. Pollock, Xin-Ming Chen

**Affiliations:** 1 Kolling Institute of Medical Research, Sydney Medical School, University of Sydney, Royal North Shore Hospital, St Leonards, Sydney, NSW 2065, Australia; 2 School of Medical Sciences (Discipline of Anatomy and Histology) – The Bosch Institute, University of Sydney, NSW 2006, Australia; 3 Australian Centre for Microscopy & Microanalysis, Madsen Building, University of Sydney, NSW 2006, Australia

## Abstract

Autophagy is emerging as an important pathway in many diseases including diabetic nephropathy. It is acknowledged that oxidative stress plays a critical role in autophagy dysfunction and diabetic nephropathy, and KCa3.1 blockade ameliorates diabetic renal fibrosis through inhibiting TGF-β1 signaling pathway. To identify the role of KCa3.1 in dysfunctional tubular autophagy in diabetic nephropathy, human proximal tubular cells (HK2) transfected with scrambled or KCa3.1 siRNAs were exposed to TGF-β1 for 48 h, then autophagosome formation, the autophagy marker LC3, signaling molecules PI3K, Akt and mTOR, and oxidative stress marker nitrotyrosine were examined respectively. *In vivo*, LC3, nitrotyrosine and phosphorylated mTOR were examined in kidneys of diabetic KCa3.1+/+ and KCa3.1−/− mice. The results demonstrated that TGF-β1 increased the formation of autophagic vacuoles, LC3 expression, and phosphorylation of PI3K, Akt and mTOR in scrambled siRNA transfected HK2 cells compared to control cells, which was reversed in KCa3.1 siRNA transfected HK2 cells. *In vivo*, expression of LC3 and nitrotyrosine, and phosphorylation of mTOR were significantly increased in kidneys of diabetic KCa3.1+/+ mice compared to non-diabetic mice, which were attenuated in kidneys of diabetic KCa3.1−/− mice. These results suggest that KCa3.1 activation contributes to dysfunctional tubular autophagy in diabetic nephropathy through PI3K/Akt/mTOR signaling pathways.

Autophagy, a term derived from the Greek, means self (auto)-eating (phagy). It is a lysosomal protein degradation pathway in cells, playing a crucial role in removing protein aggregates as well as damaged or excess organelles to maintain intracellular homeostasis and cell integrity^1^. Autophagy is mediated by a unique organelle called the autophagosome, which engulfs a portion of cytoplasm. Autophagy has two major roles: to recycle intracellular energy resources in response to nutrient-depleted conditions and to remove cytotoxic proteins and organelles under stressful conditions^2^. Three types of autophagy have been recognized in cells: macroautophagy, microautophagy, and chaperone-mediated autophagy; with different mechanisms and functions^3^. Of these three types of autophagy, “macroautophagy” is most prevalent and hereafter is referred to as “autophagy”. A basal level of autophagy is essential for cellular maintenance, differentiation, development and homeostasis, promoting the turnover of macromolecules and organelles via the lysosomal degradative pathway^4^. Several signaling pathways have been reported to regulate autophagy such as the phosphatidylinositol 3-phosphate kinase (PI3K)/Akt/mammalian target of rapamycin (mTOR) pathway^5^. In response to stress conditions including cell starvation, growth factor deprivation, hypoxia, and oxidant injury, autophagy can be induced as a cellular reaction to exert a protective role by promoting cell survival, allowing temporary cellular adaptation to unfavorable conditions^6^. Dysfunctional autophagy is involved in the pathogenesis of a variety of diseases including cancer^7^, cardiomyopathy^8^, renal disorders^9^,^10^, neurodegenerative disorders^11^,^12^, and diabetes^13^. Hence, autophagy is emerging as an important pathway in many biological processes and diseases including diabetic nephropathy^14^. However, little was known about the connection between tubular autophagy and diabetic nephropathy, thus the establishment of a linkage between tubular autophagy and diabetic nephropathy remains to be elucidated.

The intermediate-conductance calcium-activated potassium channel KCa3.1 (also known as also known as IK1, SK4 or KCNN4) is a member of the calcium-activated potassium channel (KCa) family. KCa3.1 regulates K^+^ efflux, increasing the driving force for Ca^2+^ entry through hyperpolarization of the plasma membrane^15^. It has been shown that KCa3.1 is a potential molecular target for pharmacological intervention in vascular restenosis, urinary incontinence, prostate cancer, and autoimmune disease^16^,^17^,^18^. Recently, we have demonstrated that blockade of KCa3.1 ameliorates renal fibrosis in diabetic mice through inhibition of the TGF-β1 signaling pathway^19^. We also demonstrated a key role of KCa3.1 in mediating TGF-β1 induced MCP-1 expression in renal proximal tubular cells via Smad3, p38 and ERK1/2 MAPK signaling pathways^20^. In addition, our studies demonstrated that blocking the KCa3.1 channel inhibits the NF-κB pathway, resulting in downregulation of the inflammatory marker chemokine (C-C motif) ligand 20^21^. Our studies have further shown that blockade of KCa3.1 is likely to exert its anti-fibrotic effects through inhibition of fibroblast activation^22^. Collectively, the data demonstrate that blockade of KCa3.1 is able to prevent the development of diabetic nephropathy in mouse models of diabetes mellitus. Therefore, the therapeutic potential of targeting KCa3.1 in diabetic nephropathy deserves further exploration.

It has been well documented that KCa3.1 is widely expressed throughout the body, including in erythrocytes, platelets, T and B cells, mast cells, monocytes/macrophages, microglia, epithelia, vascular endothelial cells, fibroblasts and vascular smooth muscle cells. KCa3.1 regulates Ca^2+^ entry and modulates Ca^2+^ signaling in these cells^16^,^23^,^24^,^25^,^26^. Both increased Ca^2+^ influx from the extracellular space and Ca^2+^ release from intracellular organelles leads to an increase in cytosolic calcium. It is well established that elevations in intracellular Ca^2+^ inhibits autophagy^27^. The basal autophagic flux is negatively regulated by IP3R-dependent Ca^2+^ release from the endoplasmic reticulum and cytosolic Ca^2+^ elevation maintains an increase in mTORC1 activity through AMPK independent pathways^28^. The influx of extracellular Ca^2+^ leads to mTOR activation^29^. Activation of mTOR pathway in turn leads to inhibition of autophagy. Calcium-permeable ion channels have emerged as important regulators of autophagy and the effect of such regulation is likely to depend on Ca^2+^ signals in a spatially restricted subcellular domains^30^. Since KCa3.1 regulates Ca^2+^ entry, it is proposed that there are interactions between KCa3.1 and autophagy. Therefore, in this study we investigated the role of KCa3.1 in tubular autophagy in diabetic nephropathy using *in vitro* cultured human proximal tubular cells exposed to TGF-β1 and in a validated mouse model of diabetic nephropathy^19^. Our results demonstrate that blockade of KCa3.1 was able to reverse diabetes inhibited tubular autophagy, which was mediated through inhibition of the activation of PI3K/Akt/mTOR signaling pathways.

## Results

### KCa3.1 gene silencing reversed TGF-β1-induced inhibition of tubular autophagy

**Figure 1:**
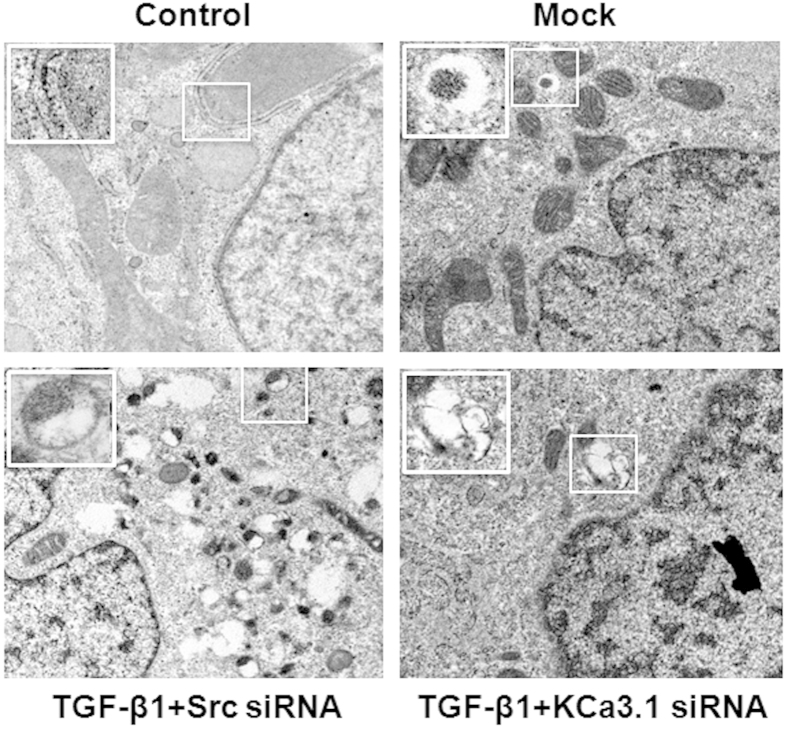
Electron microscopic evaluation of autophagy in TGF-β1-exposed HK2 cells. HK2 cells transfected with scrambled siRNA or KCa3.1 siRNA were exposed to TGF-β1 for 48 h. Representative electron micrographs show autophagic vacuoles in HK2 cells (x12000).

To determine whether KCa3.1 is involved in dysfunctional tubular autophagy, autophagy was examined in human kidney tubular cells exposed to TGF-β1 with or without KCa3.1 gene silencing. We initially used transmission electron microscopy (TEM) to monitor the appearance of autophagosomes. As shown in Fig. 1, no obvious autophagic vacuoles were found in control and mock control HK2 cells. However, numerous autophagic vacuoles appeared in TGF-β1 exposed HK2 cells transfected with control siRNA, while fewer autophagic vacuoles were observed in TGF-β1 exposed HK2 cells transfected with KCa3.1 siRNA.

**Figure 2:**
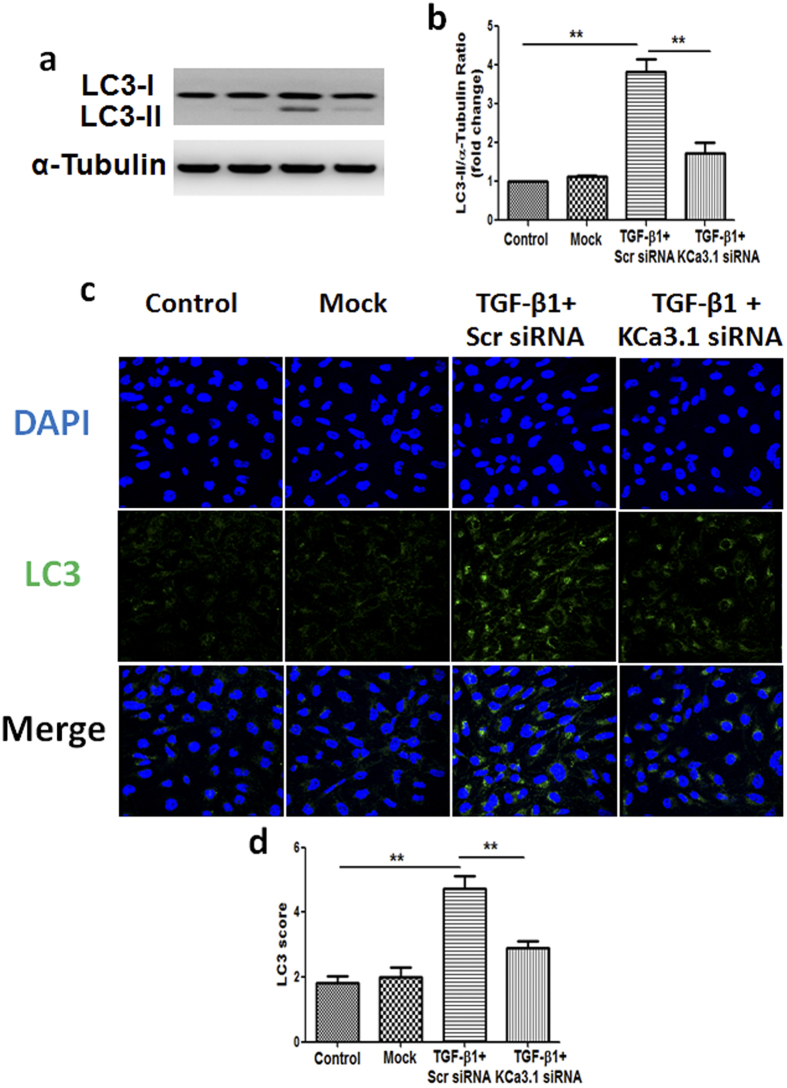
Effect of knockdown KCa3.1 on the expression of LC3 in TGF-β1-exposed HK2 cells. HK2 cells transfected with scrambled siRNA or KCa3.1 siRNA were exposed to TGF-β1 for 48 h. Immunoblot analysis of LC3 with or without KCa3.1 siRNA transfection in TGF-β1-exposed HK2 cells (**a**). Quantification of LC3 immunoblot expression in TGF-β1-exposed HK2 cells (**b**). Immunofluorescence staining of LC3 in TGF-β1-exposed HK2 cells transfected with or without KCa3.1 siRNA (**c**). Quantification of LC3 immunofluorescence expression in TGF-β1-exposed HK2 cells (**d**). Results are presented as mean + SEM. **P < 0.01. N = 4. Original magnification: ×600.

LC3, a marker of autophagy, was examined by western blot analysis. Consistent with TEM results, western blot analysis revealed that the level of LC3 was significantly increased in HK2 cells exposed to TGF-β1, while KCa3.1 silencing suppressed the TGF-β1-induced LC3 expression (*P* < 0.01, Fig. 2a,b). Immunofluorescence staining further confirmed TGF-β1-induced increased LC3 was reversed by KCa3.1 silencing (*P* < 0.01, Fig. 2c,d).

**Figure 3:**
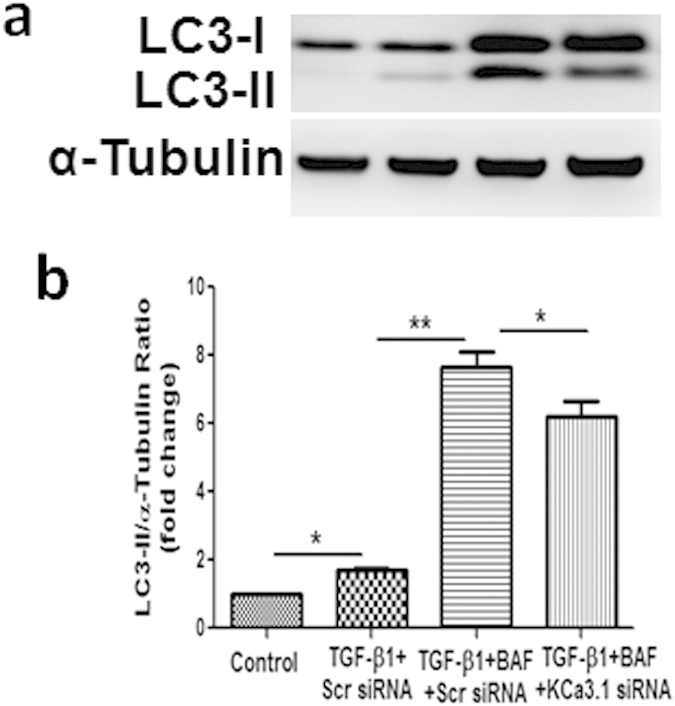
Effect of knockdown KCa3.1 on the expression of LC3 in TGF-β1-exposed HK2 cells together with or without Bafilomycin A1. HK2 cells transfected with scrambled siRNA or KCa3.1 siRNA overnight were pretreated with 50 nmol/L Bafilomycin A_1_ for 3 hours and then incubated with TGF-β1 (2 ng/ml) for 48 hours. Immunoblot analysis of LC3 with or without KCa3.1 siRNA transfection in TGF-β1-exposed HK2 cells together with Bafilomycin A_1_ (**a**). Quantification of LC3 expression in TGF-β1-treated HK2 cells together with Bafilomycin A_1_ (**b**). Results are presented as mean + SEM. *P < 0.05 and **P < 0.01. N = 4.

LC3 can accumulate due to increased upstream autophagosome formation or impaired downstream autophagosome-lysosome fusion. To distinguish between these two possibilities, we assayed LC3 in the presence of TGF-β1 plus Bafilomycin A1, which blocks downstream autophagosome-lysosome fusion. As shown in Fig. 3a, TGF-β1 significantly increased the level of LC3 as compared with control group. Treatment with Bafilomycin A1 further increased the level of LC3 in HK2 cells exposed to TGF-β1, which was partially reversed by KCa3.1 gene silencing (*P* < 0.05, Fig. 3b). These data confirmed that TGF-β1 inhibited autophagosome clearance, thereby inhibited autophagy, which was restored by blocking KCa3.1.

### Blockade of KCa3.1 reversed diabetes-induced upregulation of LC3 expression in kidney proximal tubules of diabetic mice

**Figure 4:**
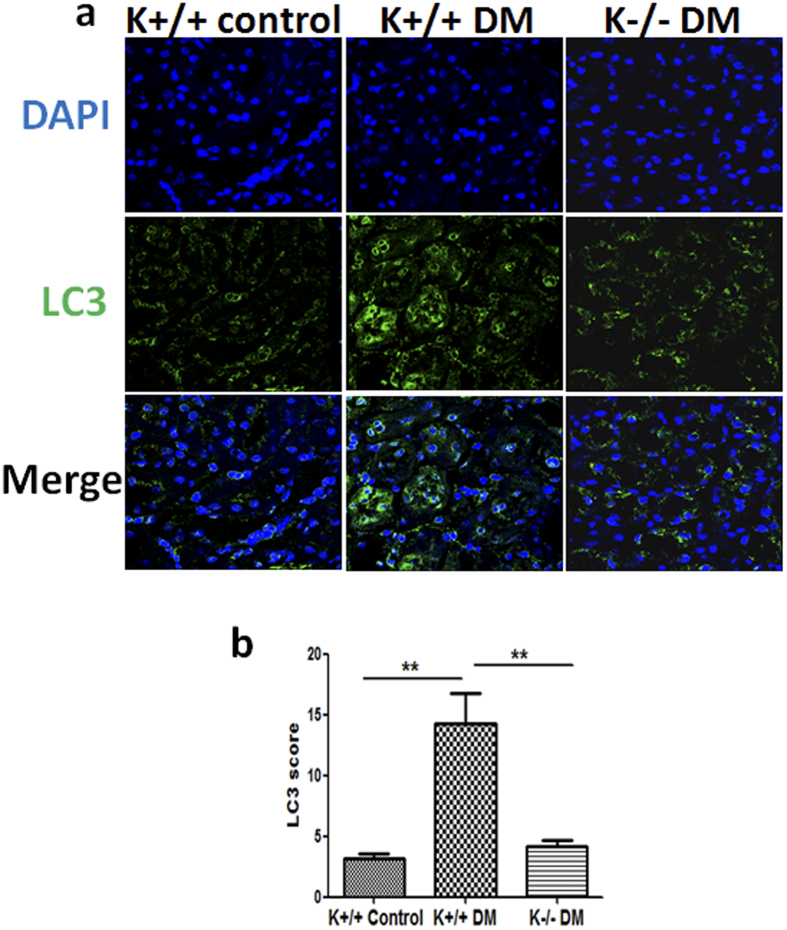
The expression of LC3 in STZ-induced diabetic KCa3.1+/+ and KCa3.1−/−mice. KCa3.1+/+ and KCa3.1−/− mice were injected with STZ to induce diabetes or citrate buffer alone as non-diabetic controls. After 24 weeks diabetes, kidney tissues were collected for immunostaining. Immunofluorescence staining of LC3 in mice kidney tissues (**a**). Quantification of LC3 expression in mice kidney tissues (**b**). Results are presented as mean + SEM. **P < 0.01. N = 8. Original magnification: ×600.

To further determine the role of KCa3.1 in regulating tubular autophagy in diabetic kidneys, autophagy marker LC3 expression was assessed in the kidneys of diabetic animals using confocal microscopy. As shown in Fig. 4a, significantly increased LC3 expression was found in kidney proximal tubules of diabetic KCa3.1+/+ mice (K+/+ DM) compared to non-diabetic mice (K+/+ control). KCa3.1 deficiency significantly inhibited diabetes-induced upregulation of LC3 expression in kidney proximal tubules of diabetic KCa3.1−/− mice (K−/− DM) (Fig. 4b), indicating that blockade of KCa3.1 reversed diabetes-induced inhibition of tubular autophagy in diabetic mice kidneys.

### KCa3.1 gene silencing inhibited TGF-β1-induced activation of PI3K/Akt/mTOR signaling pathways

**Figure 5:**
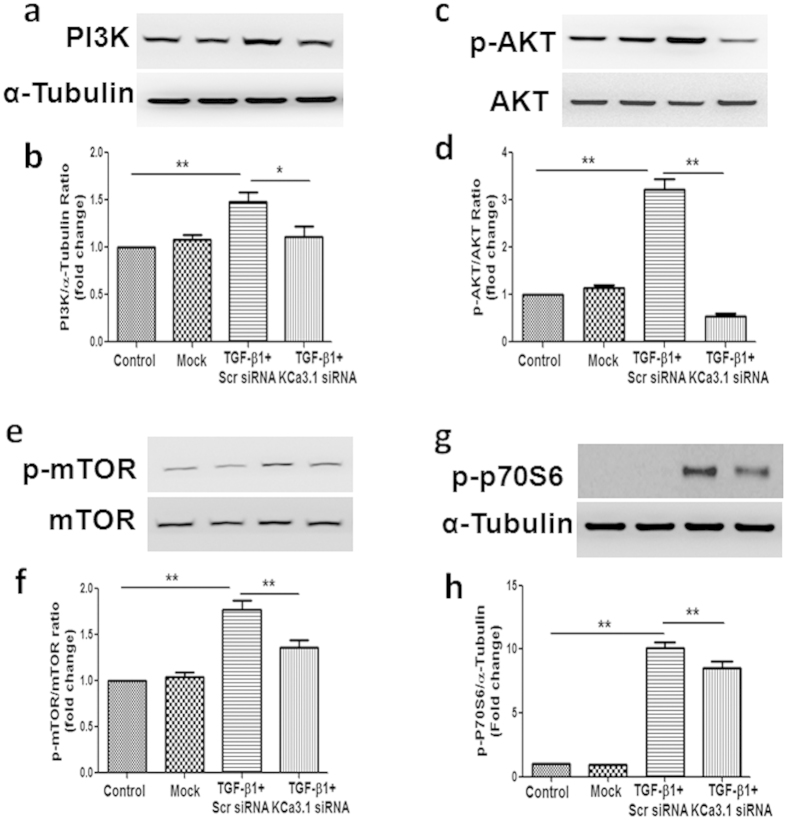
Effect of knockdown KCa3.1 on the expression of PI3K and the phosphorylation of Akt, mTOR and P70S6 in TGF-β1-stimulated HK2 cells. HK2 cells were transfected with scrambled siRNA or KCa3.1 siRNA and then incubated with TGF-β1 (2 ng/ml) for 48 h. The expression of PI3K (**a**) and phosphorylation of Akt (**c**), mTOR (**e**) and P70S6 (**g**) were determined by western blot analysis. Quantification of PI3K (**b**), Akt (**d**), mTOR (**f**) and P70S6 (**h**) expression in TGF-β1-exposed HK2 cells. Results are presented as means + SEM. *P < 0.05 and **P < 0.01. N = 4.

The PI3K/Akt/mTOR signaling pathway is one of the major pathways regulating autophagy. To understand the molecular mechanisms whereby KCa3.1 gene silencing mediates autophagy in human kidney tubular cells, we examined the effects of KCa3.1 on PI3K/Akt/mTOR signaling pathways. The expression of PI3K and the phosphorylation of Akt, mTOR were examined by western blot analysis as well as the phosphorylation of P70S6, the downstream target of mTOR. Exposure of HK2 cells to TGF-β1 resulted in significantly increased expression of PI3K and the phosphorylation of Akt, mTOR and P70S6 (*P* < 0.05, Fig. 5). Co-incubation of HK2 cells with KCa3.1 siRNA suppressed TGF-β1 induced upregulation of PI3K and activation of Akt, mTOR and P70S6 in HK2 cells (*P* < 0.05, Fig. 5). These results indicate that KCa3.1 regulation of tubular autophagy is through PI3K/Akt/mTOR signaling pathways.

### Inhibition of PI3K/Akt/mTOR signaling pathways by mTOR inhibitor Rapamycin suppressed TGF-β1-induced activation of mTOR, P70S6 and upregulation of LC3 expression

**Figure 6:**
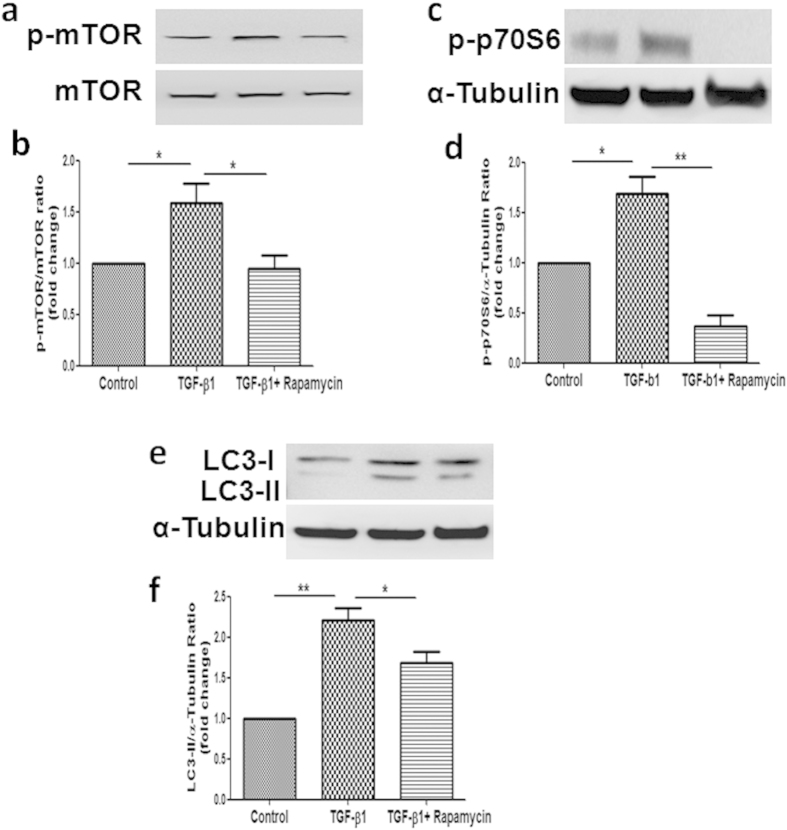
Effects of mTOR inhibitor Rapamycin on the activation of mTOR, P70S6 and the expression of LC3 in TGF-β1-stimulated HK2 cells. HK2 cells exposed to TGF-β1 (2 ng/ml) with or without Rapamycin (100 nm) for 48 h. The activation of mTOR (**a**) and P70S6 (**c**) and the expression of LC3 (**e**) were determined by Western Blot. Quantification of mTOR (**b**), P70S6 (**d**) and LC3 (**f**) expression in TGF-β1-exposed HK2 cells together with or without Rapamycin. Results are presented as means + SEM. * *P* < 0.05, N = 4.

It is well know that PI3K/Akt regulates autophagy mainly through the modulation of mTOR activity. To determine the functional significance of PI3K/Akt/mTOR activation on TGF-β1-induced inhibition of autophagy, we then tested the effect of TGF-β1 on autophagy in the presence of Rapamycin, a specific inhibitor of mTOR. As shown in Fig. 6a,b, Co-incubation with Rapamycin significantly suppressed TGF-β1 induced activation of mTOR, which was further confirmed by the inhibition of P70S6, the direct substrate of mTOR (Fig. 6c,d). Treatment with Rapamycin reversed TGF-ß1-induced upregulation of LC3 expression (Fig. 6e,f). It is important to note that, TGF-β1 activated mTOR activity was suppressed by Rapamycin, which correlates well with the induction of tubular autophagy, suggesting that the mTOR is the key effector molecule within the PI3K/Akt/mTOR pathways for TGF-β1-induced inhibition of tubular autophagy.

### KCa3.1 mediated diabetes-induced dysfunction of tubular autophagy through mTOR signaling pathway in diabetic kidneys

**Figure 7:**
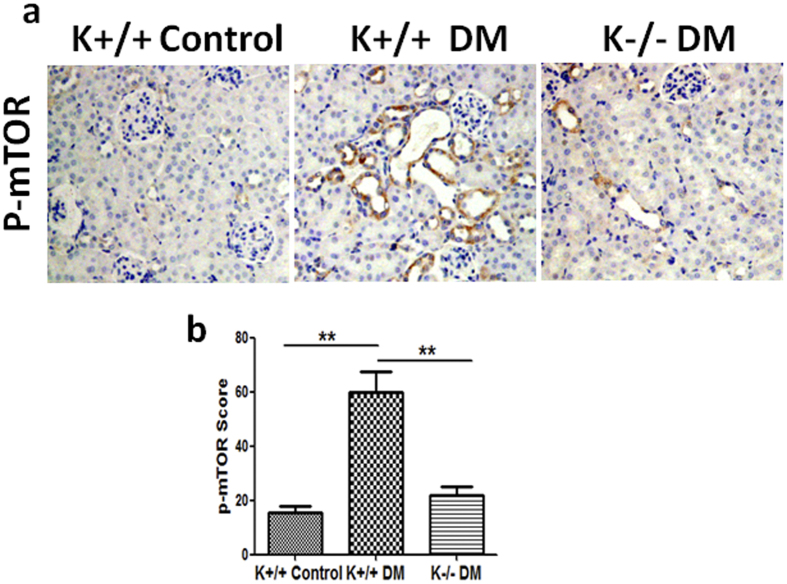
The activation of mTOR in STZ-induced diabetic KCa3.1+/+ and KCa3.1−/−mice. Immunohistochemical analysis showed increased phosphorylated mTOR in diabetic KCa3.1+/+ kidneys compared to control mice and reversed activation of mTOR in diabetic KCa3.1−/− kidneys (**a**). The quantification of phosphorylated mTOR expression in mice kidneys (**b**). Results are presented as mean + SEM. *P < 0.05 and **P < 0.01. N = 8. Original magnification: ×200.

To further determine whether mTOR signaling is an essential intermediary in KCa3.1 mediated diabetes-induced dysfunction of tubular autophagy, phosphorylation of mTOR was examined in diabetic mice kidneys. Immunohistochemical staining results showed that mTOR signaling was strongly activated in diabetic KCa3.1+/+ mice (K+/+ DM) compared to non-diabetic control mice (K+/+ control) (*P* < 0.01, Fig. 7). However, the activation was inhibited in KCa3.1 deficient diabetic mice (K−/− DM) (*P* < 0.01, Fig. 7). These data suggest that KCa3.1 mediated diabetes-induced dysfunction of tubular autophagy occurs through mTOR signaling pathway, which further confirms that mTOR is the central molecule in the PI3K/Akt/mTOR signaling pathways.

### KCa3.1 mediated diabetes-induced dysfunction of tubular autophagy through oxidative stress

**Figure 8:**
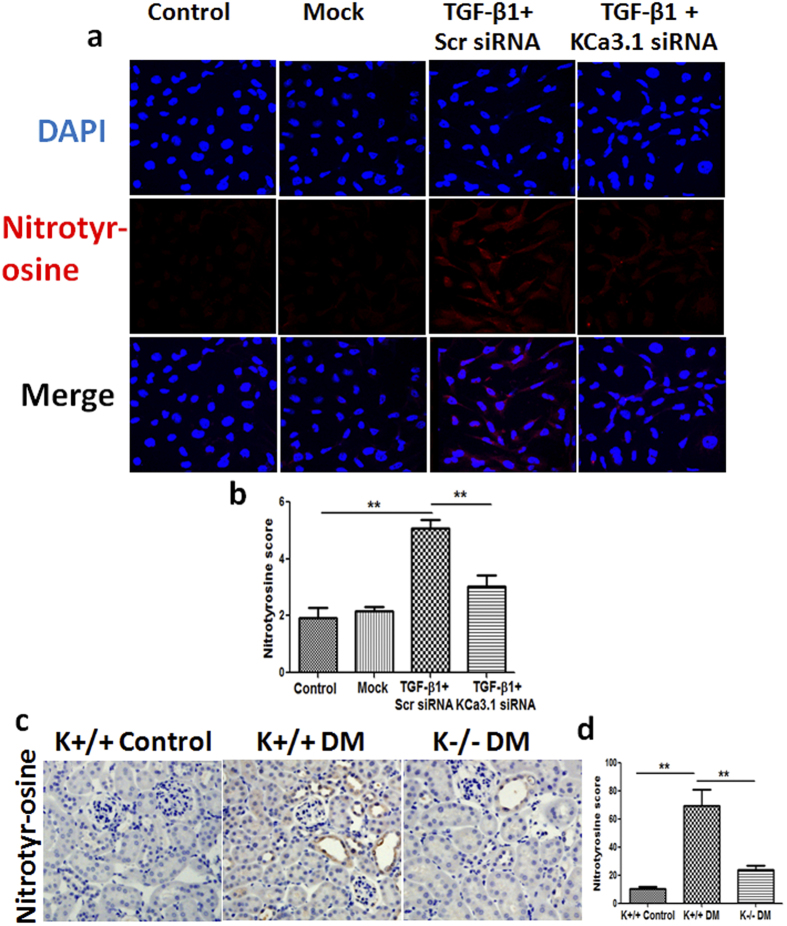
Effect of knockdown KCa3.1 on the expression of nitrotyrosine in TGF-β1-stimulated HK2 cells and the expression of nitrotyrosine in STZ-induced diabetic KCa3.1+/+ and KCa3.1−/−mice. HK2 cells transfected with scrambled siRNA or KCa3.1 siRNA were untreated or treated with TGF-β1 for 48 h. Immunofluorescence staining of nitrotyrosine in TGF-β1-exposed HK2 cells transfected with or without KCa3.1 siRNA (**a**). Quantification of nitrotyrosine expression in TGF-β1-exposed HK2 cells (**b**). N = 4. KCa3.1+/+ and KCa3.1−/− mice were injected with STZ to induce diabetes or citrate buffer alone as non-diabetic control. After 24 weeks diabetes, kidney tissues were collected for immunostaining. Immunohistochemical staining of nitrotyrosine in mice kidney tissues (**c**). Quantification of nitrotyrosine expression in mice kidney tissues (**d**). N = 8. Results are presented as mean + SEM. *P < 0.05 and **P < 0.01. Original magnification: ×600.

Recently, it has been reported that autophagy is modulated by multiple intracellular stresses including oxidative stress^31^. To investigate whether KCa3.1-mediated oxidative stress is involved in regulation of autophagy, nitrotyrosine^32^, a marker of NO-dependent oxidative stress, was examined in TGF-β1-exposed HK2 cells *in vitro*. As shown in Fig. 8a,b, TGF-β1 significantly increased the expression of nitrotyrosine in HK2 cells that was reversed by KCa3.1 silencing (*P* < 0.01). Furthermore, we examined the expression of nitrotyrosine in diabetic mice kidneys. Immnunohistochemical staining results confirmed that a marked induction of nitrotyrosine was observed in the kidneys of diabetic KCa3.1+/+ animals when compared with non-diabetic controls, which were reversed in the kidneys of diabetic KCa3.1−/− mice (*P* < 0.01, Fig. 8c,d). Together, these results indicate that KCa3.1 is likely to mediate dysregulation of tubular autophagy through oxidative stress.

## Discussion

This study was undertaken to define the role of KCa3.1 in regulating tubular autophagy in diabetic nephropathy. Our study demonstrated that impaired autophagy was found in cultured human kidney proximal tubular cells exposed to TGF-β1 as well as in kidney proximal tubular cells of diabetic mice. Blockade of KCa3.1 using siRNA technology, reduced formation of autophagosomes in tubular cells exposed to TGF-β1. KCa3.1 gene silencing also partially reversed BafilomycinA1 blockade of autophagosome clearance, indicating that KCa3.1 was involved in dysfunctional tubular autophagy in diabetic condition, which was further confirmed in diabetic KCa3.1−/− mouse model. In addition, our results show that KCa3.1 mediated autophagy was dependent on the activation of the PI3K/Akt/mTOR signaling pathways while mTOR is the central molecule in the PI3K/Akt/mTOR signaling pathways. Furthermore, KCa3.1 mediated dysregulation of tubular autophagy is likely to be associated with oxidative stress.

Autophagy has been observed in various parts of kidney including the proximal tubule, which has a central role in the pathogenesis of diabetic nephropathy. Numerous studies have reported on autophagy of proximal tubular cells in models of acute renal injury. Autophagy is readily induced by renal ischemia–reperfusion and cisplatin damage to the kidneys^9^,^33^. Reduced autophagic activity worsens acute kidney injury, suggesting that stress-inducible autophagy occurs as a renoprotective phenomenon^34^,^35^. In addition, mice lacking proximal tubular epithelial cell-specific Atg5, an important modulator in the expansion steps of autophagosome development, have been found to develop renal tubular injury with age^9^,^34^. However, to date, the role of tubular autophagy in diabetic nephropathy has not been defined.

As TGF-β1 plays an important role in diabetic nephropathy, in this study we used TGF-β1 to mimic diabetic condition in the *in vitro* experiments. To investigate whether impaired tubular autophagy was involved in diabetic nephropathy, electron microscopy, the gold standard to monitor the formation of autophagosomes, was used to demonstrate the accumulation of autophagic vacuoles in TGF-β1-exposed human proximal tubular cells. The results were further confirmed by an increased LC3 level detected by western blotting and immunofluorescence staining. Since increased LC3 levels can be associated with either enhanced autophagosome synthesis or reduced autophagosome turnover. To better interpret changes in levels of processed LC3, Bafilomycin A1, an inhibitor of autophagosomal fusion with lysosomes, that inhibit degradation of autolysosome content and lead to the accumulation of autophagosome, were used in the study. Bafilomycin A1, further increased TGF-β1-induced LC3 level in HK2 cells, suggesting that the autophagic flux is impaired in TGF-β1-exposed human proximal tubular cells. The results (Figs 2 and 3) are consistent with our findings in diabetic kidneys (Fig. 4). Thus our results indicate that the inhibition of tubular autophagy is associated with diabetic nephropathy. However, it should be noted that TGF-β1 plays a multifunctional role in autophagy. TGF-β1 could induce autophagy or inhibit autophagy by activation of the mammalian target of mTOR via PI3K/Akt signaling pathways^36^. Therefore, TGF-β1 may exert both stimulatory and inhibitory effects on authophagy, which may depend on the specific cell type and context in which it is studied^36^.

We have previously shown that blockade of KCa3.1 reversed diabetic-induced upregulation of inflammatory and fibrotic responses through a TGF-β1/Smad dependent signaling pathway^19^. Since TGF-β1 induced expression of KCa3.1 and suppressed autophagy in renal tubular cells, the link between KCa3.1 and autophagy in renal tubular cells was characterized. Our results demonstrated that TGF-β1-induced expression of LC3, and accumulation of autophagic vacuoles were decreased significantly in KCa3.1 gene silenced renal tubular cells exposed to TGF-β1 with or without Bafilomycin A1 (Figs 2 and 3). *In vivo* studies also confirmed that KCa3.1 gene knockdown reversed diabetes induced upregulation of LC3 expression compared to diabetic control mice, indicating that blockade of KCa3.1 promoted tubular autophagosome clearance, which was inhibited in diabetic control mice (Fig. 4). There data suggested that restoration of ‘normal’ autophagy may be a key mechanism by which blockade of KCa3.1 ameliorates diabetic nephropathy. To date, the mechanism of TGF-β1 and KCa3.1 on LC3 expression is not fully understood. However, as we have shown previously, TGF-β1 upregulated KCa3.1 expression in renal tubular cells^19^ and it is well reported that KCa3.1 promotes Ca^2+^ influx from extracellular space and Ca^2+^ release from intracellular organelles, which subsequently inhibits autophagic flux by preventing the fusion between autophagosomes and lysosomes^37^,^38^. This study has shown that TGF-β1 inhibited autophagic flux which led to the accumulation of LC3. KCa3.1 gene silencing may prevent TGF-β1-induced Ca^2+^ influx and maintain autophagic flux, thus reversed TGF-β1 induced LC3 expression.

The PI3K/Akt/mTOR signaling pathway is a well-known pathway involved in the regulation of autophagy. PI3K/Akt regulates autophagy mainly through the modulation of mTOR activity. mTOR is an evolutionarily conserved protein kinase and forms two functional complexes, termed mTORC1 and mTOR complex 2^39^. mTORC1 has been shown to be a negative regulator of autophagy by integrating signals that are emitted by growth factors, amino acids, glucose, and energy status^2^. Recent studies suggest that the pathogenesis of diabetic nephropathy is associated with impaired autophagic activity via activation of the mTOR pathway^40^,^41^,^42^. In this study, we demonstrated that the activation of PI3K/Akt/mTOR is essential for the effect of KCa3.1 in mediating autophagy. Our results show that suppression of KCa3.1 with siRNA gene silencing blocked TGF-β1-induced activation of PI3K, Akt and downstream signaling of mTOR, which led to the induction of autophagy in HK2 cells. This is consistent with the finding that inhibition of PI3k/Akt/mTOR with Rapamycin, the specific inhibitor of mTOR, reversed TGF-β1 inhibited autophagy. Collectively, these data suggest that KCa3.1 mediated autophagy is depended on the PI3K/Akt/mTOR pathways in TGF-β1-stimulated HK2 cells. This is further supported by the *in vivo* results that KCa3.1 deficiency reversed diabetes suppressed autophagy through inhibition of the activation of the PI3K/Akt/mTOR signaling pathways in diabetic mice.

Oxidative stress, resulting from excessive production or accumulation of reactive oxygen species (ROS), is a major modulator of autophagy. ROS has been shown to play a major role in the development of diabetic nephropathy^43^,^44^,^45^. Recent studies suggested that autophagy might be able to ameliorate diabetic nephropathy by reducing oxidative stress^46^,^47^. In this study, we have shown increased oxidative stress in TGF-β1-exposed HK2 cells as well as in diabetic kidneys, which was correlated with the inhibition of autophagy. Blockade of KCa3.1 not only reduced ROS production but also induced autophagy in both *in vitro* and *in vivo* diabetic models. These results suggested that inhibition of KCa3.1 may elicit an antifibrotic effect at least in part mediated through induction of autophagy by reducing the excess ROS production.

In summary, our studies from both *in vitro* and *in vivo* experimental models provide evidence that tubular autophagy was inhibited in diabetic nephropathy. Blockade of KCa3.1 reversed diabetes-induced inhibition of autophagy, which was dependent on the PI3K/Akt/mTOR signaling pathways. This study thus provides new insights, suggesting the renoprotective effect of KCa3.1 blockade in diabetic nephropathy is at least partly due to restoration of dysfunctional tubular autophagy.

## Materials and Methods

### Materials

Lipofectamine 2000 and tissue culture medium were provided from Invitrogen Life Technologies (Carlsbad, CA). Anti-LC3-B antibody was purchased from Abcam (Cambridge, MA) and anti-α-tubulin antibody, Bafilomycin A1 and Rapamycin were from Sigma (St. Louis, MO). Anti-PI3K, anti-phospho-Akt, anti-Akt, anti-phospho-mTOR, anti-mTOR, anti-phospho-P70S6 and horseradish peroxidase-conjugated secondary antibodies were purchased from Cell Signaling Technology (Danvers, MA). Anti-nitrotyrosine antibody was from Millipore (Darmstadt, Germany). Alexa488-conjugated secondary antibodies were obtained from Invitrogen (Carlsbad, CA).

### Cell culture and KCa3.1 gene silencing

Immortalized human proximal tubular cells (HK2 cells) were obtained from ATCC (Manassas, VA). HK2 cells were grown in keratinocyte serum-free media medium (Invitrogen, CA) and used for experiment at passages 5–15.

HK2 cells were transfected with either siRNA targeting KCa3.1 or scrambled control siRNA using Lipofectamine 2000 reagent (Invitrogen, CA) according to the manufacturer’s instructions. The targeting siRNA sequence for KCa3.1 is 5′-GCACCUUUCAGACACACUU-3′ (GenePharma, Shanghai). After transfection overnight, the cells were incubated with TGF-β1 (2 ng/ml) for 48 hours, cell lysates and total RNA were collected for further analysis. Alternatively, cells were pretreated with 50 nmol/L Bafilomycin A_1_ for 3 hours and then incubated with TGF-β1 for 48 hours. To evaluate the effects of mTOR inhibition, HK2 cells were exposed to TGF-β1 (2 ng/ml) with or without Rapamycin (100 nm) for 48 hours.

### The measurement of autophagy using transmission electron microscopy

HK2 cells grown on glass coverslips were transfected with KCa3.1 or scrambled siRNA overnight and then incubated with TGF-β1 for 48 hours as described above. The samples were prepared for transmission electron microscopic analysis as previously reported^48^. Briefly, cells were washed with pre-warmed PBS twice, and then fixed in 2% glutaraldehyde in PBS for 60 minutes at room temperature. Fixed cells were washed with PBS 3 times then postfixed with 1% in osmium tetroxide in PBS for 1hr. After rinsing 3 times with distilled water, the samples were further stained with 1% tannic acid for 1 hour. Finally, the cells were infiltrated and double-embedded in Epon. Sections of 70 nm were generated with an ultramicrotome (Ultracut 7, Leica) and post-stained with 2% aqueous uranyl acetate and Reynold’s lead citrate for 10 min each. The specimens were examined with the JEOL 2100TEM at 200 kV.

### Immunocytofluorescence staining

For indirect immunofluorescence, cells cultured on glass coverslips were washed with PBS, fixed with 4% formaldehyde at room temperature for 15 min, PBS wash once for 5 min and then permeabilized in 0.3% Triton X-100 in PBS for 10 min,and blocked with 2% bovine serum albumin (BSA) in PBS for 1 h at room temperature. Cells were incubated with primary antibodies against LC3 and nitrotyrosine in 2% BSA in PBS overnight at 4 °C. After washing with PBS, cells were incubated with secondary anti-rabbit Alexa Fluor-488 or anti-rabbit Alexa Fluor-633 (Invitrogen) for 40 min at room temperature. Cells were then washed with PBS and counterstained with 4′, 6-diamidino-2 phenylindole (DAPI)-mounting medium (Invitrogen). The immunofluorescence images were collected by Confocal fluorescence microscopy (Leica Microsystems, Mannheim, Germany).

### Western blotting

Equal amount of cell lysate samples were separated by SDS-PAGE, and then transferred to Hybond ECL nitrocellulose membrane (Amersham, USA). The membranes were incubated with primary antibodies LC3, PI3k, Akt, mTOR, P70S6 and Tubulin at 4 °C overnight followed with HRP-conjugated secondary antibody (Amersham, USA). The blots were then detected with standard ECL technique, and the bands were quantified by densitometry using LAS-4000 Imaging System (FUJIFILM, Japan).

### Animal studies

KCa3.1−/− mice were kindly provided by Dr. James Melvin, National Institute of Dental and Craniofacial Research, Bethesda, MD, USA. Eight-week-old male KCa3.1+/+ mice and KCa3.1−/− mice weighing approximately 20 to 25 g were assigned to receive either 55 mg/kg of streptozotocin (STZ) (Sigma, MO) diluted in 0.1 M citrate buffer, pH 4.5, or citrate buffer alone by intraperitoneal injection as described previously^19^. All animals were housed in the Kearns Animal Facility of Kolling Institute of Medical Research with a stable environment maintained at 22 ± 1 °C with a 12/12-h light-dark cycle.

Mice were weighed and their blood glucose levels measured using the Accu-chek glucometer (Roche Diagnostics) weekly and only STZ-treated animals with blood glucose >16 mmol/l were considered diabetic. After animals were culled, left kidneys were removed and snap frozen for the isolation of RNA or protein, and right kidneys were perfused with PBS and fixed in 10% buffered formalin for histological examination.

This study was carried out in strict accordance with the recommendations in the Guide of the National Health and Medical Research Council of Australia’s Code for the Care and Use of Animals for Scientific Purposes. The protocol was approved by the Animal Research Ethics Committee of Royal North Shore Hospital (Permit Number: 1101–001A).

### Immunostaining

Frozen tissues were fixed in ice-cold acetone for 10 mins and then washed twice with ice cold PBS. After pre-incubation with 2% BSA in PBS for 1 hour, the tissues were incubated with primary antibodies against LC3 for 1 hour at room temperature. After washing with PBS, cells were incubated with secondary anti-rabbit Alexa Fluor-488 (Invitrogen) for 40 min at room temperature. Cells were then washed with PBS and counterstained with 4′, 6-diamidino-2 phenylindole (DAPI)-mounting medium (Invitrogen). The immunofluorescence images were collected by confocal fluorescence microscopy (Leica Microsystems, Mannheim, Germany).

Paraffin-embedded kidney sections were used for immunohistochemical staining. Briefly, after heat retrieval, endogenous peroxidase activity was blocked by incubation in 0.3% hydrogen peroxide. After pre-incubation with 10% protein block (Dako, CA) for 10 minutes at room temperature to block nonspecific binding of antibodies, the tissues were incubated overnight at 4 °C with primary antibodies against nitrotyrosine and p-mTOR. After incubation with appropriate secondary antibodies, sections were developed with 3, 3-diaminobenzidine (Dako, CA) to produce a brown color and counterstained with haematoxylin. Positive signals in the renal cortex regions were quantified using Image J software as previously described^19^.

### Statistical analysis

Results from at least four independent experiments were expressed as mean ± SEM. Statistical analysis of data from two groups was compared by two-tail t-test. Data from multiple groups was performed by one-way ANOVA, followed by Tukey post test. Statistical significance was determined as *P* < 0.05.

## Additional Information

**How to cite this article**: Huang, C. *et al.* KCa3.1 mediates dysfunction of tubular autophagy in diabetic kidneys via PI3k/Akt/mTOR signaling pathways. *Sci. Rep.* **6**, 23884; doi: 10.1038/srep23884 (2016).

## References

[b1] Mizushima N., Levine B., Cuervo A. M. & Klionsky D. J. Autophagy fights disease through cellular self-digestion. Nature. 451, 1069–1075 (2008). 18305538 10.1038/nature06639 PMC2670399

[b2] Tanaka Y. *et al.* Autophagy as a therapeutic target in diabetic nephropathy. Exp Diabetes Res. 2012, 628978 (2012). 22028701 10.1155/2012/628978 PMC3199112

[b3] Massey A. C., Zhang C. & Cuervo A. M. Chaperone-mediated autophagy in aging and disease. Curr. Top. Dev. Biol. 73, 205–235 (2006). 16782460 10.1016/S0070-2153(05)73007-6

[b4] Ravikumar B. *et al.* Regulation of mammalian autophagy in physiology and pathophysiology. Physiol. Rev. 90, 1383–1435 (2010). 20959619 10.1152/physrev.00030.2009

[b5] Inoki K. mTOR signaling in autophagy regulation in the kidney. Semin. Nephrol. 34, 2–8 (2014). 24485024 10.1016/j.semnephrol.2013.11.002 PMC4911697

[b6] Del Principe D., Lista P., Malorni W. & Giammarioli A. M. Fibroblast autophagy in fibrotic disorders. J. Pathol. 229, 208–220 (2013). 23018629 10.1002/path.4115

[b7] Chen N. & Karantza-Wadsworth V. Role and regulation of autophagy in cancer. Biochim. Biophys. Acta. 1793, 1516–1523 (2009). 19167434 10.1016/j.bbamcr.2008.12.013 PMC3155287

[b8] Nakai A. *et al.* The role of autophagy in cardiomyocytes in the basal state and in response to hemodynamic stress. Nat. Med. 13, 619–624 (2007). 17450150 10.1038/nm1574

[b9] Kimura T. *et al.* Autophagy protects the proximal tubule from degeneration and acute ischemic injury. J. Am. Soc. Nephrol. 22, 902–913 (2011). 21493778 10.1681/ASN.2010070705 PMC3083312

[b10] Kaushal G. P. Autophagy protects proximal tubular cells from injury and apoptosis. Kidney Int. 82, 1250–1253 (2012). 23203020 10.1038/ki.2012.337 PMC4068008

[b11] Hara T. *et al.* Suppression of basal autophagy in neural cells causes neurodegenerative disease in mice. Nature. 441, 885–889 (2006). 16625204 10.1038/nature04724

[b12] Komatsu M. *et al.* Loss of autophagy in the central nervous system causes neurodegeneration in mice. Nature. 441, 880–884 (2006). 16625205 10.1038/nature04723

[b13] Gonzalez C. D. *et al.* The emerging role of autophagy in the pathophysiology of diabetes mellitus. Autophagy. 7, 2–11 (2011). 20935516 10.4161/auto.7.1.13044 PMC3359481

[b14] Ding Y. & Choi M. E. Autophagy in diabetic nephropathy. J. Endocrinol. 224, R15–30 (2015). 25349246 10.1530/JOE-14-0437 PMC4238413

[b15] Begenisich T. *et al.* Physiological roles of the intermediate conductance, Ca^2+^-activated potassium channel Kcnn4. J. Biol. Chem. 279, 47681–47687 (2004). 15347667 10.1074/jbc.M409627200

[b16] Kohler R. *et al.* Blockade of the intermediate-conductance calcium-activated potassium channel as a new therapeutic strategy for restenosis. Circulation. 108, 1119–1125 (2003). 12939222 10.1161/01.CIR.0000086464.04719.DD

[b17] Wulff H., Kolski-Andreaco A., Sankaranarayanan A., Sabatier J. M. & Shakkottai V. Modulators of small- and intermediate-conductance calcium-activated potassium channels and their therapeutic indications. Curr. Med. Chem. 14, 1437–1457 (2007). 17584055 10.2174/092986707780831186

[b18] Ohya S. *et al.* Malignancy grade-dependent expression of K+-channel subtypes in human prostate cancer. J. Pharmacol. Sci. 109, 148–151 (2009). 19129683 10.1254/jphs.08208sc

[b19] Huang C. *et al.* Blockade of KCa3.1 ameliorates renal fibrosis through the TGF-beta1/Smad pathway in diabetic mice. Diabetes. 62, 2923–2934 (2013). 23656889 10.2337/db13-0135 PMC3717839

[b20] Huang C., Day M. L., Poronnik P., Pollock C. A. & Chen X. M. Inhibition of KCa3.1 suppresses TGF-beta1 induced MCP-1 expression in human proximal tubular cells through Smad3, p38 and ERK1/2 signaling pathways. Int. J. Biochem. Cell Biol. 47, 1–10 (2014). 24291552 10.1016/j.biocel.2013.11.017

[b21] Huang C., Pollock C. A. & Chen X. M. High glucose induces CCL20 in proximal tubular cells via activation of the KCa3.1 channel. PLos One. 9, e95173 (2014). 24733189 10.1371/journal.pone.0095173 PMC3986377

[b22] Huang C. *et al.* KCa3.1 mediates activation of fibroblasts in diabetic renal interstitial fibrosis. Nephrol. Dial. Transplant. 29, 313–324 (2014). 24166472 10.1093/ndt/gft431 PMC3910344

[b23] Wulff H. & Kohler R. Endothelial small-conductance and intermediate-conductance KCa channels: an update on their pharmacology and usefulness as cardiovascular targets. J. Cardiovasc. Pharmacol. 61, 102–112 (2013). 23107876 10.1097/FJC.0b013e318279ba20 PMC3565027

[b24] Ghanshani S. *et al.* Up-regulation of the IKCa1 potassium channel during T-cell activation. Molecular mechanism and functional consequences. J. Biol. Chem. 275, 37137–37149 (2000). 10961988 10.1074/jbc.M003941200

[b25] Tan C. Y. *et al.* Thioredoxin-interacting protein: a potential therapeutic target for treatment of progressive fibrosis in diabetic nephropathy. Nephron. 129, 109–127 (2015). 25662516 10.1159/000368238

[b26] Pena T. L., Chen S. H., Konieczny S. F. & Rane S. G. Ras/MEK/ERK Up-regulation of the fibroblast KCa channel FIK is a common mechanism for basic fibroblast growth factor and transforming growth factor-beta suppression of myogenesis. J. Biol. Chem. 275, 13677–13682 (2000). 10788486 10.1074/jbc.275.18.13677

[b27] Reidy K., Kang H. M., Hostetter T. & Susztak K. Molecular mechanisms of diabetic kidney disease. J. Clin. Invest. 124, 2333–2340 (2014). 24892707 10.1172/JCI72271 PMC4089448

[b28] Devi T. S. *et al.* TXNIP links innate host defense mechanisms to oxidative stress and inflammation in retinal Muller glia under chronic hyperglycemia: implications for diabetic retinopathy. Exp Diabetes Res. 2012, 438238 (2012). 22474421 10.1155/2012/438238 PMC3313582

[b29] Mahmood D. F., Abderrazak A., El Hadri K., Simmet T. & Rouis M. The thioredoxin system as a therapeutic target in human health and disease. Antioxid Redox Signal. 19, 1266–1303 (2013). 23244617 10.1089/ars.2012.4757

[b30] Chen J., Jing G., Xu G. & Shalev A. Thioredoxin-interacting protein stimulates its own expression via a positive feedback loop. Mol. Endocrinol. 28, 674–680 (2014). 24628418 10.1210/me.2014-1041 PMC4004782

[b31] Koya D. *et al.* Effects of antioxidants in diabetes-induced oxidative stress in the glomeruli of diabetic rats. J. Am. Soc. Nephrol. 14, S250–253 (2003). 12874441 10.1097/01.asn.0000077412.07578.44

[b32] Pacher P., Obrosova I. G., Mabley J. G. & Szabo C. Role of nitrosative stress and peroxynitrite in the pathogenesis of diabetic complications. Emerging new therapeutical strategies. Curr. Med. Chem. 12, 267–275 (2005). 15723618 10.2174/0929867053363207 PMC2225483

[b33] Jiang M. *et al.* Autophagy in proximal tubules protects against acute kidney injury. Kidney Int. 82, 1271–1283 (2012). 22854643 10.1038/ki.2012.261 PMC3491167

[b34] Liu S. *et al.* Autophagy plays a critical role in kidney tubule maintenance, aging and ischemia-reperfusion injury. Autophagy. 8, 826–837 (2012). 22617445 10.4161/auto.19419

[b35] Takahashi A. *et al.* Autophagy guards against cisplatin-induced acute kidney injury. Am. J. Pathol. 180, 517–525 (2012). 22265049 10.1016/j.ajpath.2011.11.001

[b36] Ding Y. & Choi M. E. Regulation of autophagy by TGF-beta: emerging role in kidney fibrosis. Semin. Nephrol. 34, 62–71 (2014). 24485031 10.1016/j.semnephrol.2013.11.009 PMC3912517

[b37] Park H. W. *et al.* Pharmacological correction of obesity-induced autophagy arrest using calcium channel blockers. Nature communications. 5, 4834 (2014). 10.1038/ncomms5834 PMC4157315 25189398

[b38] Tian X. *et al.* A voltage-gated calcium channel regulates lysosomal fusion with endosomes and autophagosomes and is required for neuronal homeostasis. PLoS biology. 13, e1002103 (2015). 25811491 10.1371/journal.pbio.1002103 PMC4374850

[b39] Zoncu R., Efeyan A. & Sabatini D. M. mTOR: from growth signal integration to cancer, diabetes and ageing. Nat. Rev. Mol. Cell Biol. 12, 21–35 (2011). 21157483 10.1038/nrm3025 PMC3390257

[b40] Yamahara K. *et al.* Obesity-mediated autophagy insufficiency exacerbates proteinuria-induced tubulointerstitial lesions. J. Am. Soc. Nephrol. 24, 1769–1781 (2013). 24092929 10.1681/ASN.2012111080 PMC3810079

[b41] Godel M. *et al.* Role of mTOR in podocyte function and diabetic nephropathy in humans and mice. J. Clin. Invest. 121, 2197–2209 (2011). 21606591 10.1172/JCI44774 PMC3104746

[b42] Inoki K. *et al.* mTORC1 activation in podocytes is a critical step in the development of diabetic nephropathy in mice. J. Clin. Invest. 121, 2181–2196 (2011). 21606597 10.1172/JCI44771 PMC3104745

[b43] Ha H., Hwang I. A., Park J. H. & Lee H. B. Role of reactive oxygen species in the pathogenesis of diabetic nephropathy. Diabetes Res. Clin. Pract. 82 Suppl 1, S42–45 (2008). 18845352 10.1016/j.diabres.2008.09.017

[b44] Ha H. & Lee H. B. Reactive oxygen species and matrix remodeling in diabetic kidney. J. Am. Soc. Nephrol. 14, S246–249 (2003). 12874440 10.1097/01.asn.0000077411.98742.54

[b45] Kashihara N., Haruna Y., Kondeti V. K. & Kanwar Y. S. Oxidative stress in diabetic nephropathy. Curr. Med. Chem. 17, 4256–4269 (2010). 20939814 10.2174/092986710793348581 PMC3708695

[b46] Xu Y. *et al.* Resveratrol protects against hyperglycemia-induced oxidative damage to mitochondria by activating SIRT1 in rat mesangial cells. Toxicol. Appl. Pharmacol. 259, 395–401 (2012). 22015446 10.1016/j.taap.2011.09.028

[b47] Mori J. *et al.* Angiotensin 1–7 mediates renoprotection against diabetic nephropathy by reducing oxidative stress, inflammation, and lipotoxicity. American journal of physiology. Renal physiology. 306, F812–821 (2014). 24553436 10.1152/ajprenal.00655.2013

[b48] Huang C. *et al.* Thioredoxin-interacting protein mediates dysfunction of tubular autophagy in diabetic kidneys through inhibiting autophagic flux. Lab. Invest. 94, 309–320 (2014). 24492284 10.1038/labinvest.2014.2

